# Development and validation of a sexual relations satisfaction scale in patients with breast cancer — “SEXSAT-Q”

**DOI:** 10.1186/s12955-019-1197-7

**Published:** 2019-08-17

**Authors:** Rosario González Mancha, Montserrat Muñoz, Luis de la Cruz-Merino, Lourdes Calvo, Josefina Cruz, Jose Manuel Baena-Cañada, Yolanda Fernandez, Manuel Ramos, Cesar Augusto Rodriguez, Jose Ignacio Chacón, Isabel Palomero, Julia Llinares, María Rivero, Miguel Ángel Ruiz

**Affiliations:** 1 Medical Oncology Department, Hospital Universitario Virgen del Rocío, Seville, Spain; 2 Medical Oncology Department, Hospital Universitario Clínic —, Barcelona, Spain; 3 Medical Oncology Department, Hospital Universitario Virgen de la Macarena, Seville, Spain; 4 Medical Oncology Department, Complejo Hospitalario Universitarios A Coruña (CHUAC), A Coruña, Spain; 5 Medical Oncology Department, Hospital Universitario de Canarias, Santa Cruz de Tenerife, Spain; 6 Medical Oncology Department, Hospital Puerta del Mar, Cádiz, Spain; 7 Medical Oncology Department, Hospital Universitario Central de Asturias, Oviedo, Spain; 8 Medical Oncology Department, Centro Oncológico de Galicia, A Coruña, Spain; 9 Medical Oncology Department, Hospital Clínico Universitario de Salamanca — IBSAL, Salamanca, Spain; 10 Medical Oncology Department, Hospital Universitario Virgen de la Salud, Toledo, Spain; 11 Medical Oncology Department, Hospital Universitario Gregorio Marañón, Madrid, Spain; 12 Laboratorios Pfizer España, Avenida de Europa, 20 – B- Parque Empresarial La Moraleja, 28108 Alcobendas (Madrid), Spain; 13 Psychology Department, Universidad Autónoma de Madrid, Madrid, Spain

**Keywords:** Breast, Cancer, Sexual, Satisfaction, Dysfunction, Questionnaire

## Abstract

**Purpose:**

Because the currently available questionnaires to evaluate sexual changes on breast cancer women only address the sexual sphere with a few questions our purpose was to develop a questionnaire that assesses changes in sexual dysfunction and satisfaction in women treated for breast cancer.

**Methods:**

A sample was selected of women aged between 18 and 65 who had had surgery for breast cancer, completed neoadjuvant/adjuvant chemotherapy treatment and could be receiving adjuvant hormonal treatment, with an active sex life at least 3 months before starting treatment. Metastatic disease was excluded. A questionnaire structured in 4 dimensions was developed. The MOS SF-12 and QLQ-BR23 questionnaires were also provided. The following metric properties were evaluated: item analysis; internal consistency; temporal stability; construct validity; concurrent, convergent and divergent validity; and feasibility.

**Results:**

Three samples were recruited: a pilot sample of 20; a reduction sample of 152; and a validation sample of 148. The presence of 6 dimensions was confirmed: 1) Loss of sex drive; 2) worsening of body image; 3) psychological coping; 4) discomfort during intercourse; 5) satisfaction with sexual relations; and 6) satisfaction with breast reconstruction. Good goodness-of-fit statistics were obtained (χ^2^/df = 1.5, GFI = 0.9, AGFI = 0.84, CFI = 0.959, RMSEA = 0.062). Reliability was good (*α = 0.855*), as was test–retest stability (*r = 0.838*). The correlation with the convergent questionnaires proved to be higher than that obtained with generic measurements.

**Conclusions:**

We were able to develop a short questionnaire (17 items) capable of measuring sexual satisfaction in women with breast cancer with good metric properties.

**Electronic supplementary material:**

The online version of this article (10.1186/s12955-019-1197-7) contains supplementary material, which is available to authorized users.

## Introduction

Being diagnosed with cancer brings changes to an individual’s personal, family, social, professional and financial life and to their sexual relations with their partner [1]. In addition, a person’s body image can be altered by some surgical treatments which mainly affect an individual’s psychological and sexual sphere [2, 3]. This situation can be made even worse by extended courses of treatment. Most patients feel an additional loss, beyond the disease itself, and find it both hard to accept and difficult to discuss in the clinic. Moreover, the doctor often devotes little time to asking about changes experienced in sexuality.

In view of the above we decided that breast cancer would be an appropriate disease in which to conduct a study on sexual dysfunction, owing to its high incidence and variability of age, geographic location, level of education, etc. [4, 5]. The aim of this study was to develop and validate an understandable and easy to complete questionnaire (“Sexual Satisfaction Questionnaire” (SEXSAT-Q)), which covered the issues of concern of the patients and was capable of distinguishing what their main problem was for use in the Spanish population of women with breast cancer (Additional file 1: Sexual Satisfaction Questionnaire). The currently available questionnaires are quite limited and only address the sexual sphere with a few questions and with no questions exploring sexual satisfaction [6, 7].

In 2016 a meta-analysis was published that includes a systematic review of published studies addressing the sexual dysfunction in women with various cancer types (endometrial cancer, cervical cancer, vulvar cancer, breast cancer, etc.) using a validated diagnostic tool. In this meta-analysis, eleven studies referred to breast cancer were included [8]. The authors concluded that women with cancer showed low index scores with a high prevalence of sexual dysfunction.

In the general population, a questionnaire was developed to assess women sexual function suggesting important gender differences in the patterning of female sexual function in comparison with similar questionnaire studies in males [9].

Although breast cancer is the most frequent cancer in women and the second most common cancer in the world (25% of all cancers) [10]. A specific questionnaire that measures changes in sexual dysfunction and satisfaction in these women treated for breast cancer has not been developed.

## Patients and methods

### Patients

Between October 2009 and July 2011 we enrolled female outpatients with breast cancer, aged between 18 and 65, with active sex lives at least 3 months before starting treatment, who could had undergone conservative surgery or mastectomy with or without immediate or delayed reconstruction. They had completed neoadjuvant/adjuvant chemotherapy at least 3 months and up to 3 years beforehand and they were allowed to be receiving hormone treatment. Women who were exclusively receiving adjuvant hormonal therapy had completed at least one year and no more than 3 years of treatment. The patients had to be able to understand and answer the study questionnaires. Patients with metastatic disease were excluded. All of the patients signed the informed consent form.

Patients were analysed by, time since last period on enrolment in the study, regardless of age, ovarian reserve and whether or not they were receiving hormone treatment. We defined as postmenopausal women with 12 or more months of amenorrhoea, perimenopausal women with less than 12 months of amenorrhoea and premenopausal women who continued to have normal menses.

In each participating centre, the researcher performed a random probabilistic sampling by sequential recruitment according to the attention demand among those patients who come to consultation and meet the inclusion criteria. There were 4 samples; a pilot sample with 20 patients enrolled; a reduction sample with 156 patients enrolled; a validation sample with 148 patients and a sub-sample of 61 patients at the re-test sample.

The pilot sample consisted of 20 patients recruited in a randomized manner by the participating researchers. This sample was used to detect problems in understanding and/or reading the suggested questions and to collect comments from patients. Only 3 sites participated in the recruitment of the pilot sample (Virgen del Rocio, Virgen de la Macarena, Clinic i Provincial). For the reduction sample, 156 patients who fulfilled selection criteria were included. In this sample a wider questionnaire was delivered allowing to eliminate the questions that were not understood as relevant or that did not discriminate the samples. This selection was random and sequential, covering the pre-determined quotas. In the recruitment of this sample all sites participated. The validation sample consisted of 148 additional patients. This sample completed the Case Report Form (CRF) that included: the reduced questionnaire, the validation questionnaires, and the clinical evaluation of the responsible researcher. All sites participated in the recruitment of the reduction sample. The reduced questionnaire was provided for a second time to a subsample of 61 patients with an allowed time window from 1 week to 15 days to check the temporary stability of the measurements without mediating changes in the clinical intervention.

### Methodology

We designed an observational, cross-sectional, multicentre study performed along routine clinical practice therefore, without interfering with the patients’ regular treatment. The study was developed by 11 Spanish Breast Cancer Oncologists in 11 Spanish sites. Every physician selected a sample of patients that met the inclusion criteria in their daily clinical practice. The study was supervised by a panel of experts made up of 3 clinical investigators and one methodologist and approved by the ethics committees of all the participating sites. Every patient received information about the objective of the study, the expected benefits and the voluntary nature of the study, and gave its informed consent prior to their inclusion.

We extracted written testimonies provided by patients with breast cancer and compiled the experience of the investigators in their clinical practice to identify the dimensions and items to be included in the questionnaire and 4 dimensions of interest were identified: 1) Sexual dysfunction; 2) Dissatisfaction with self-image; 3) Anxiety generated by the treatment; and 4) Overall rating of quality of sexual activity.

### The study involved 4 phases

#### *Pilot sample,* to check item legibility and comprehensibility

*Reduction of the Questionnaire,* to check whether the patients’ responses were in line with the structure proposed, to assess the metric properties of the items and to reduce the questionnaire.

The reduction of the questionnaire and the determination of the dimensions were accomplished through a sequence of exploratory factor analyses and an internal consistency analysis. Principal Components and Principal axis were used; and Varimax (orthogonal) and Oblimin (oblique) rotations were performed. K1 rule [11, 12], percentage of explained variance and size of the eigenvalues after rotation [13] were studied to determine the number of factors given the known behaviour to underestimate or overestimate the correct number of factors [14]. Internal consistency was assessed using Cronbach’s alpha and alpha change when removing items.

The questionnaire’s length was reduced following Gorsuch and Russell proposals [15–17]. Firstly, items with a clear floor or ceiling effect (more than 50% of responses in the first or last response categories) were discarded. Next, an exploratory factor analysis was conducted with the 26 items of the scale to determine the number of underlying factors. Lastly, the dimensionality and internal consistency [18] of each subscale was analysed, assuming unidimensionality.

*Validation;* including the questionnaire in a case report form together with relevant clinical information, socio-demographic information and additional instruments: MOS SF-12, QLQ-BR23, and EuroQol visual analogue scale.

*Re-test sample:* 61 patients who completed the reduced questionnaire a second time a week later to estimate test–retest reliability.

The following metric properties were studied: (1) *feasibility*: administration time, floor and ceiling effects, and percentage of missing values for each item; (2) *reliability*: internal consistency [19] and item-total correlation; test–retest reliability (Pearson correlation) and intraclass correlation coefficient [20]; (3) *content validity*: this was ensured by the expert group and by consultation with the patients; (4) *construct validity*: Confirmatory factor analysis was applied; (5) *concurrent validity*: SEXSAT-Q scores were correlated with SF-12, QLQ-BR23, and EuroQol VAS; (6) *discriminant validity*: items were assessed to discriminate between patients in Q1 and Q4. The following goodness-of-fit criteria were used [21]: χ^2^ (*p > 0.05*), χ^2^/df ≤ 2, GFI ≥ 0.9, AGFI> 0.9, CFI > 0.9, RMSEA< 0.08).

All the statistical analyses were carried out using IBM SPSS 20 and AMOS 20 software.

## Instruments

### Perceived health status visual analogue scale

A visual analogue scale included in the EuroQol questionnaire to assess the patient’s current health status [22]. The scale is graduated and marked at intervals of ten, running from 0 (worst imaginable health status) to 100 (best imaginable health status).

### Quality of life

Spanish version of the generic SF-12 quality of life questionnaire validated in Spain [23–25]. This questionnaire is self-administered with supervision and has 12 items and two summary components: physical health and mental health.

### Cancer-specific quality of life questionnaire

Spanish version of the EORTC QLQ- BR-23 cancer-specific quality-of-life questionnaire [26]. This questionnaire has 23 items that measure 5 dimensions: side effects of therapy; arm symptoms; breast symptoms; body image; and sexual functioning. It also has 2 items assessing sexuality and future perspective.

## Results

### Pilot sample

All patients completed the questionnaire properly and the average response time was 5 min 40s. Compiled comments referred to font size, difficulties in properly interpreting time from disease diagnosis and relevance of questions related to reconstructive surgery. A question relating satisfaction with current sexual relations was added, leading to the long version of the questionnaire with 24 questions.

### Reduction of the questionnaire

The reduction sample was composed of 152 assessable women (see Table 1).

**Table 1:** Socio-demographic description of the reduction and validation samples

	Reduction Sample *N* = 152	Validation Sample *N* = 148
Age (years): Mean (SD)	49.5 (7.8)	47.9 (8.4)
Studies (%)		
Primary	39.1	28.9
Secondary	31.1	33.8
Graduate	29.8	37.3
Actual couple: Yes (%)	98.7	97.9
Relationship (years): Mean (SD)	23.7 (11.5)	24.1 (10.2)
Contraceptive: Yes (%)	31.3	46.9
Climacteric stage (%)		
Pre-menopause	12.6	25.3
Perimenopause	14.6	14.4
Post-menopause	72.8	60.3
Breast-Conserving Surgery (%)		58.3
Mastectomy (%)		42.9
Severity (%)		
Stage I		47.3
Stage II		42.5
Stage III		9.5
Adjuvant Chemotherapy (%)		85.5
Duration (month): Mean (SD)		5 (2.87)
Adjuvant Hormonotherapy (%)		82.6
Duration (month); Mean (SD)		50.4 (NR)
Complementary Radiotherapy (%)		81.5

The questionnaire was well understood and accepted by patients. Most of the questions showed no ceiling or floor effects. Overall, 84% of patients thought that the questionnaire was suitable or very suitable for assessing their personal situation.

The exploratory factor analysis resulted in a 5-dimensional solution, accounting for 71% of available variance (see Table 2): 1) loss of sex drive; 2) worsening of body image; 3) psychological coping; 4) discomfort during intercourse; and 5) satisfaction with sexual relations.

**Table 2:** Exploratory factor analysis estimated factor loadings (each dimension isolated)

	Factor					
	1	2	3	4	5	6
Q1. Sexual relations satisfactory before	0.566					
Q10. Sexual relations satisfactory during tx	0.850					
Q14. Sexual relations satisfactory currently	0.860					
Q11. Sex life has been pushed into the background		0.824				
Q12. Decreased sex drive since diag.		0.908				
Q13. I have more trouble reaching orgasm since diag.		0.899				
Q7. Embarrassed to show my body			0.761			
Q8. I have trouble looking in the mirror and accepting myself			0.525			
Q9. My body image has worsened			0.751			
Q2. Distressed since diagnosis				0.796		
Q3. Depressed since tx				0.812		
Q6. More tired than before				0.800		
Q4. Fear of pain during sex					0.838	
Q5. Pain owing to vaginal dryness					0.838	
Q22. Satisfaction with surgery						.772
Q23. Surgery improved sexual relations						.845
Q24. Trust Surgery will improve sexual relations						.878
First Eigenvalue	1.783	2.312	1.825	1.93	1.403	2.081
Accounted Variance (%)	59.44	77.07	60.82	64.44	70.17	69.67

Reliability statistics for the full scale were good, α = 0.871 (Table 3). Three dimensions obtained high values: effects of menopause, body image and psychological coping. The discomfort during sex dimension showed modest reliability and ended up with 2 items. The satisfaction with sexual relations dimension had higher reliability, but also did not reach a value of 0.7. The scale referring to breast reconstruction was only relevant for 20 patients.

**Table 3:** Reliability

	Cronbach’s α Reduction	Cronbach’s α Validation	95% ICC		Test–Retest r	Items
			Lower	Upper		
Satisfaction with sexual relations	0.659	0.711	0.619	0.784	0.862	3
Loss of sex drive	0.801	0.876	0.836	0.908	0.781	3
Body image	0.861	0.856	0.810	0.892	0.808	3
Psychological coping	0.840	0.774	0.701	0.831	0.762	3
Discomfort during intercourse	0.553	0.517	0.328	0.654	0.868	2
Reconstruction	0.772	0.701	0.371	0.873	^a^	3
Full scale	0.871	0.855	0.816	0.889	0.838	14

^a^Not available

The final questionnaire was made up of 17 items organised into 6 dimensions. The reconstruction dimension was separated from the others.

### Validation

The validation sample consisted of 148 patients (see Table 1).

**Fig. 1:**
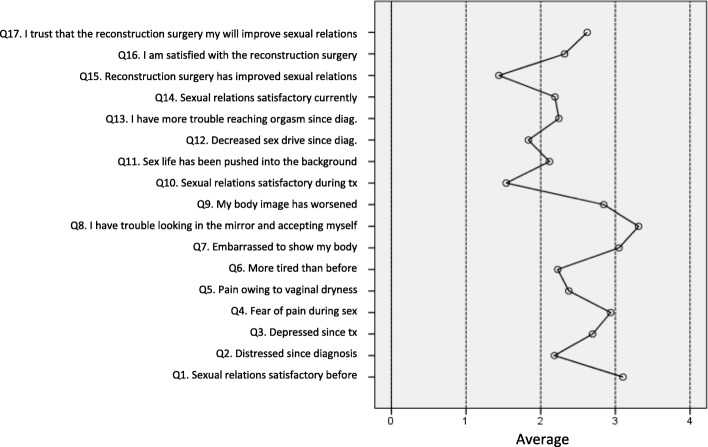
Average score on the questionnaire questions (Minimum = 0, Maximum = 4). The higher the score, the higher the satisfaction

Blank responses did not exceed 4%. None of the items showed a floor or ceiling effect except items 4 and 8, with 54 and 62% of responses in the lower category, respectively (see Fig. 1 showing the mean scores for each item).

Items that did not distinguishing between Q1 and Q4 groups were item 1 (it was left as a comparison for questions 10 and 14) and item 15 (the effective sample size for it was very small).

**Fig. 2:**
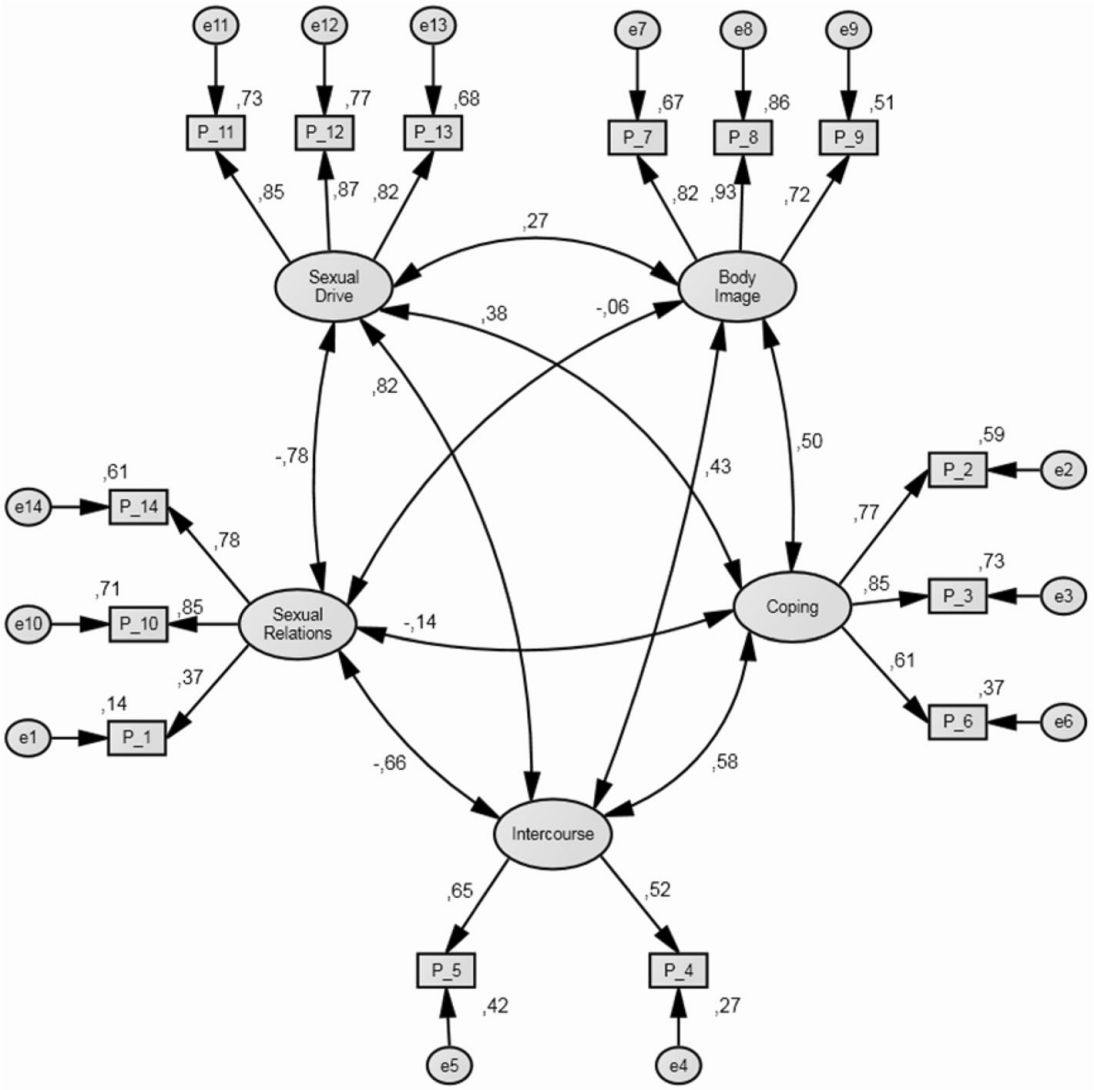
Confirmatory factor analysis maximum likelihood estimates. Note: Boxes represent observed variables and circles un-observed error variables. One-headed lines represent regression effects and the values above them, raw estimated regression weights, two-headed arrows represent correlations between latent variables. Values above exogenous variables represent variances

Confirmatory factor analysis attained a stable solution (Fig. 2), with all parameters being statistically significant, and a good fit was obtained (χ^2^ = 101.5, df = 67; *p* = 0.004, χ^2^/df = 1.5, GFI = 0.9, AGFI = 0.84, CFI = 0.959, RMSEA = 0.062). All the dimensions of the questionnaire proved to be closely interrelated, except for the more psychological dimensions.

Reliability statistics for the full scale were good, with *α = 0.855* (not including the items referring to reconstruction) and test–retest *r = 0.838*. Dimension reliabilities ranged from *α = 0.517* to *α = 0.876*, with better test–retest stability (*r = 0.762 to r = 0.868)*.

Correlations of the total score with other concurrent measurements were moderate with the SF-12 physical component and mental component. Correlation with the EQ-5D VAS was somewhat higher and the highest correlation was with the QLQ-BR23 (Table 4). Correlations of the three sexual relations items in the QLQ-BR23 and the satisfaction with sexual relations dimension was r = 0.715 and with Loss of sex drive r = − 0.632.

**Table 4:** Observed correlation with other concurrent validation scales

Scale	Sex. Sat.	SF-12 PC	SF-12 MC	VAS EQ-5D	QLQ-BR23
Sexual satisfaction — Total	1				
SF-12 Physical Component	−0.387	1			
SF-12 Mental Component	−0.407	0.114†	1		
VAS EQ-5D	−0.526	0.546	0.542	1	
QLQ-BR23 total	0.608	−0.432	−0.535	−0.622	1

All correlations *p* < 0.001. † Not significant (*p* = 0.177)

The mean observed total score was 61.0, which was relatively high compared to the average score on the metric and the distribution of the scores had a slight negative skew. Table 5 shows the algorithm for calculating the scores in the dimensions along with the mean score and standard deviation for each dimension.

**Table 5:** SEXSAT scoring algorithm, number of items and descriptive statistics by dimension

Dimension	Shorthand	items	Scoring Algorithm	Mean	SD
Satisfaction with sexual relations	SR	3	SR = (X1 + X10 + X14) × 8.33	56.6	23.7
Loss of sex drive	SDr	3	SDr = 100 – (X11 + X12 + X13) × 8.33	51.1	34.4
Body image	BI	3	BI = 100 – (X7 + X8 + X9) × 8.33	76.8	27.3
Psychological coping	PCo	3	PCo = 100 – (X2 + X3 + X6) × 8.33	59.4	26.7
Discomfort during intercourse	DI	2	DI = 100 – (X4 + X5) × 12.5	66.5	30.2
Reconstruction	RE	3	RE = (X15 + X16 + X17) × 8.33	53.7	29.0
Full scale	TOT	17	TOT = (SR + SDr + BI + PCo + DI + RE) / 6	59.0	21.5
Full scale without reconstruction	TOT2	14	TOT2 = (SR + SDr + BI + PCo + DI) / 5	62.0	20.0

Note: The higher the score, the higher the quality of life. *SD* standard deviation

Comparing the mean scores on the dimensions, dimension 3 (*satisfaction with body image*) showed a significantly higher mean than the other dimensions. By contrast, the lowest means were obtained in dimension 1 (*satisfaction with sexual relations*) which were not significantly different from each other, nor were they significantly different from the average on dimension 4. When the satisfaction with breast reconstruction dimension, is considered for the patients to whom it applies, the average level of satisfaction is the lowest of all the dimensions.

## Discussion

Although there are quality-of-life questionnaires for cancer patients (EORTC-QLQ-BR23) containing the odd question relating to sexuality or self-image, no questionnaire has been developed to date that focuses entirely on this subject. The inclusion of questions concerning patients’ sexuality in the above questionnaires points to the importance of this issue as an integral part of health-related quality of life.

Analysis of the dimensionality of the scale detected 5 interrelated dimensions referring to: effects of menopause; body image; psychological coping with the disease; discomfort during intercourse; and satisfaction with sexual intercourse; plus a sixth dimension on satisfaction with breast reconstruction.

In both reduction and validation phases the questions concerning reconstruction was only relevant for those patients it applied to. This dimension needs to be taken into account separately. For this reason, we have provided two methods of correction (Table 5) for patients who have had or not reconstruction.

In the confirmatory analysis of the dimensionality of the scale, 5 interrelated dimensions were again identified, referred to satisfaction with sexual relations: decreased sex drive; body image; psychological coping with the disease; discomfort during intercourse; and discomfort during sexual relations. Furthermore, a 6th dimension on satisfaction with breast reconstruction was maintained.

The reliability of the total scale was good (α = 0.855), indicating that it is possible to obtain sufficiently accurate measurements from the construct.

With respect to validation with other scales, our scale correlated significantly with the QLQ-BR23 specific quality-of-life scale and to a somewhat lesser degree with the EuroQoL generic quality-of-life scale and the physical and mental components of health status on the SF-12. While correlations with sexual relations items in the QLQ-BR23 were high and significant. Therefore, we may affirm that our scale is found to be related to patients’ quality of life but is found to measure aspects somewhat different to quality of life itself.

Clinically, satisfaction scores are quite high, with an average exceeding the midpoint of the metric (63 out of 100). The dimension on which the least satisfaction was expressed was the one on breast reconstruction, although this dimension was only relevant for 14% of the sample.

The outcomes and survival of women with breast cancer have experienced a significant improvement in the last years comparing to the past decades [27]. This is related to the greater life expectancy and the development of new personalized therapies that improve the quality of life of these patients [28]. Nowadays women with breast cancer live longer and better, but to date the assessment of their sexual life has not been integrated into the decision making of therapeutic algorithms although it has demonstrated to impact in their quality of life [29].

This specific sexual satisfaction questionnaire in breast cancer is an easy tool for physicians that could facilitate the communications between the oncologists and breast cancer female patients and could help in the election of the most suitable treatment for each woman (Additional file 1: Sexual Satisfaction Questionnaire).

The advances in cancer management are directed towards the personalized medicine that tries to attend all the spheres of the disease including the sexual life. The integration of this questionnaire into the decision making process is a topic of increasing interest and could orientate the design of future clinical trials. It could be of great interest to compare the impact that two therapeutic approaches have in the sexual life and satisfaction of these women based on this scale, or in determining if a new drug has a negative impact in sexual life comparing to the standard treatment in a head-to-head trial. Further research is needed in order to assess the relation of sexual satisfaction with important mental health indicators (such as anxiety, depression, anger and hostility), along with their forecasting capabilities in predicting treatment acceptability and the possible need of psychological support or additional treatment.

This questionnaire to date is the only one published that focuses on sexual relations satisfaction exclusively in breast cancer patients. It can be employed as an easy tool for physicians to facilitate the communications about sexuality satisfaction between the oncologists and breast cancer female patients, integrating this dimension as an important part of the health-related quality of life of these women. An important limitation of the study is the limited sample size. The questionnaire should be validated in a larger sample to obtain additional information that could make comparisons between the individual treatments and other aspects that could be related to sexual satisfaction in this population. Aspects as sexual orientation or decrease of frequency of relationships were not included in the questionnaire, and could be also affected by the disease.

The validation process has been limited to ensure concept and structural validity. More evidences are planned to be gathered about sensitivity to change, predictive validity and known groups validity. Sexual satisfaction is conceived as very important sphere of personal, social and family roles, but it is also true that this importance varies with age, and it could even become meaningless in the elder age. From the perspective of construct validity, it would be interesting to study at what point of personal ontogenetic development sexual satisfaction becomes trivial.

## Conclusions

The obtained results show that this instrument may be used on a day-to-day basis to determine the level of satisfaction with their sexual relations of patients with breast cancer in regular medical practice, and it might even help differentiate treatments in terms of how they affect this satisfaction dimension.

Therefore we may conclude that the SEXSAT-Q questionnaire is an instrument capable of measuring sexual satisfaction in patients with breast cancer through the assessment of 5 dimensions: 1) Loss of sex drive; 2) worsening of body image; 3) psychological coping; 4) discomfort during intercourse; 5) satisfaction with sexual relations; plus an additional dimension on satisfaction with breast reconstruction (Additional file 1: Sexual Satisfaction Questionnaire).

We need to gather a larger sample to obtain additional data on validation of the breast reconstruction dimension.

## Limitations

Two dimensions present reliabilities bellow 0.8 and they should be employed with care when used isolated for diagnostic purposes.

## Additional file

Additional file 1: Sexual Satisfaction Questionnaire. (DOCX 58 kb)

## Data Availability

Please contact author for data request.

## Abbreviations

AGFI: Adjusted Goodness-of-Fit Statistic
CFI: Comparative Fit Index
EORTC: European Organisation for Research and Treatment of Cancer
EuroQol VAS: Euro Quality of Life Visual Analogue Scale
GFI: Goodness-of-fit statistic
MOS SF-12: Medical Outcomes Study - Short Form Health Survey
QLQ-BR23: Quality of Life Questionnaire-Breast Cancer Module
RMSEA: Root Mean Square Error of Approximation
SEXSAT-Q: Sexual Satisfaction Questionnaire
