# Supplementary material for: Development and validation of a sexual relations satisfaction scale in patients with breast cancer — “SEXSAT-Q”
Source: Health Qual Life Outcomes. 2019 Aug 17;17:143. doi: 10.1186/s12955-019-1197-7 (PMC6698031; doi:10.1186/s12955-019-1197-7)
Supplement: Supplementary file 1 — Sexual Satisfaction Questionnaire. (DOCX 58 kb) [file 12955_2019_1197_MOESM1_ESM.docx]

Additional file 1

**SEXUAL SATISFACTION QUESTIONNAIRE**

We would like to know your opinion on the repercussions that your breast cancer and the treatment you have received have had on your sexual relations and your relationship with your partner. We would like to ask you to answer as honestly as possible.

For each question, tick the number that best reflects your opinion. There are no right or wrong answers. If you are not sure about any of the responses, tick the one that seems most appropriate.

|  | No, not at all | To some degree | A little but  not to a great degree | To quite  a degree | Yes,  very much |
| --- | --- | --- | --- | --- | --- |
| 1. Before the diagnosis of my disease, my sexual relations  were satisfactory ................................................................ | 0 | 1 | 2 | 3 | 4 |
| 2. Since I was diagnosed, I've been feeling distressed and anxious ............................................................................... | 0 | 1 | 2 | 3 | 4 |
| 3. Since I started treatment I’ve felt sad and depressed........ | 0 | 1 | 2 | 3 | 4 |
| 4. I’m scared that during foreplay, my partner’s caresses and embraces might hurt me .................................................... | 0 | 1 | 2 | 3 | 4 |
| 5. Sexual relations are uncomfortable and/or painful owing to vaginal dryness ................................................................... | 0 | 1 | 2 | 3 | 4 |
| 6. I feel more tired and don’t feel like doing as many things as  I did before I was ill ............................................................ | 0 | 1 | 2 | 3 | 4 |
| 7. The changes I’ve experienced during treatment make me embarrassed to show my body .......................................... | 0 | 1 | 2 | 3 | 4 |
| 8. Since I started the treatment I have trouble looking in the mirror and accepting how I am now................................... | 0 | 1 | 2 | 3 | 4 |
| 9. I think the treatment has significantly worsened my body image.................................................................................. | 0 | 1 | 2 | 3 | 4 |
| 10. My sexual relations were/are satisfactory during the  treatment for my disease ................................................... | 0 | 1 | 2 | 3 | 4 |
| 11. My sex life has been pushed into the background because  of my disease...................................................................... | 0 | 1 | 2 | 3 | 4 |
| 12. My sex drive has decreased since I was diagnosed with my disease................................................................................ | 0 | 1 | 2 | 3 | 4 |
| 13. Since I was diagnosed with my disease, I have more trouble reaching orgasm .................................................... | 0 | 1 | 2 | 3 | 4 |
| 14. At the moment my sexual relations are satisfactory ....... | 0 | 1 | 2 | 3 | 4 |

Additional questions (answer these questions only if you have had or are planning to have reconstructive surgery)

|  | Not applicable | No, not at all | To some degree | A little but  not to a  great degree | To quite  a degree | Yes, Very much |
| --- | --- | --- | --- | --- | --- | --- |
| 15. The breast reconstruction surgery has improved my sexual relations................................................ | □ | 0 | 1 | 2 | 3 | 4 |
| 16. I am satisfied with the outcome of the reconstructive surgery .......................................... | □ | 0 | 1 | 2 | 3 | 4 |
| 17. I trust and hope that the breast reconstruction surgery improves my sexual relations ................... | □ | 0 | 1 | 2 | 3 | 4 |
